# Methods to Assess Bias and Pool Results of Studies of Drugs Associated With Fall-Related Injuries

**DOI:** 10.1093/geroni/igaf122.2965

**Published:** 2025-12-31

**Authors:** Jimmie Roberts, Andrew Zullo, Sarah Berry

**Affiliations:** Hebrew SeniorLife, Boston, Massachusetts, United States; Brown University, Providence, Rhode Island, United States; Hebrew SeniorLife & Harvard Medical School, Hebrew SeniorLife, Massachusetts, United States

## Abstract

Studies of older adults could benefit from an index that quantifies the cumulative effects of medications on fall outcomes, but no such evidence-based, composite outcome measure exists. Our objective was to develop an index for the burden of fall-related injury (FRI)-inducing medications while adjusting estimates to account for potential bias in the existing literature. A literature review was conducted to identify full-text studies estimating the effects of medications on FRIs from January 1990 to present. Studies were assessed for bias by a trained epidemiologist using either the Risk of Bias in Non-randomized Studies of Interventions (ROBINS-I) or the Revised Cochrane risk-of-bias (RoB) tool for randomized controlled trials (RCTs). Each effect estimate was then corrected for the degree of potential bias assessed – a correction determined by the research team (e.g., moderate bias=20% reduction). Corrected estimates were standardized, then pooled into a single summary estimate for each medication class. In total, 106 articles were identified: 75 observational studies and 5 RCTs were assessed for RoB. Among observational studies, 67 had serious RoB, 8 had moderate RoB, and none had low or critical RoB. Among RCTs, 4 had some concerns, 1 had low RoB, and none had high RoB. For bias among 5 observational studies of statins with FRIs, statins increased the risk of FRIs by 151% before and 91% after bias correction. Correcting for potential bias ensures our index is robust despite heterogeneous findings. Our approach to developing this index could be extended to assess other critical outcomes like major bleeds.

